# Case Report: Epidermal growth factor receptor germline variant associated with epilepsy and rare, distinctive cerebral MRI abnormalities

**DOI:** 10.3389/fradi.2026.1782924

**Published:** 2026-05-18

**Authors:** Elise A. Ferreira, Machteld M. Oud, Erik-Jan Kamsteeg, Clara D. M. van Karnebeek, Mirjam Langeveld, Edward M. Leter, Maartje J. Vogel, Nicole I. Wolf, Stefan D. Roosendaal, Jeroen J. van Dillen, Olaf E. M. G. Schijns, Simon Tousseyn, Christianne Hoeberigs, Rob P. W. Rouhl, Saskia N. van der Crabben

**Affiliations:** 1 Department of Paediatrics, Emma Children's Hospital, Amsterdam University Medical Center, Amsterdam, Netherlands; 2 United for Metabolic Diseases, Netherlands; 3 Department of Human Genetics, Donders Institute for Brain, Cognition and Behaviour, Radboud University Medical Center, Nijmegen, Netherlands; 4 Emma Center for Personalized Medicine, Department of Human Genetics, Amsterdam University Medical Center, Amsterdam, Netherlands; 5 Department of Endocrinology and Metabolism, Research Institute Gastroenterology, Endocrinology and Metabolism (AGEM), Amsterdam University Medical Center, Amsterdam, Netherlands; 6 Department of Clinical Genetics, Maastricht University Medical Center+, Maastricht, Netherlands; 7 Department of Human Genetics, Amsterdam University Medical Center, Amsterdam, Netherlands; 8 Amsterdam Leukodystrophy Center, Department of Child Neurology, Emma Children's Hospital, Amsterdam University Medical Center, Amsterdam, Netherlands; 9 Amsterdam Neuroscience, Cellular & Molecular Mechanisms, Vrije Universiteit, Amsterdam, Netherlands; 10 Department of Radiology and Nuclear Medicine, Amsterdam University Medical Center, Amsterdam, Netherlands; 11 Department of Neurology, Elkerliek Hospital, Helmond, Netherlands; 12 Department of Neurosurgery, Maastricht University Medical Center+, Maastricht, Netherlands; 13 Academic Center for Epileptology Kempenhaeghe/MUMC+, Heeze and Maastricht, Netherlands; 14 Mental Health and Neuroscience Research Institute, Maastricht University, Maastricht, Netherlands; 15 Department of Radiology, Maastricht University Medical Center+, Maastricht, Netherlands; 16 Department of Neurology, Maastricht University Medical Center+, Maastricht, Netherlands

**Keywords:** case report, EGFR, epilepsy, germline variant, MRI abnormalities

## Abstract

**Background:**

Somatic pathogenic variants in *EGFR* have recently been implicated in lesional focal epilepsy, typically in association with low-grade epilepsy-associated tumors. Germline *EGFR* variants, however, have not previously been linked to epilepsy-related neuroimaging phenotypes.

**Case presentation:**

We report a large multigenerational family in which multiple individuals presented with epilepsy, progressive cognitive impairment, and striking, bilateral mesiotemporal and thalamic MRI abnormalities. Through deep phenotyping and reanalysis of exome sequencing data a rare, heterozygous, germline *EGFR* variant [NM_005228.5:c.866C > A p.(Ala289Asp)] was identified and shown to segregate with the neurological phenotype.

**Conclusion:**

This case report expands the phenotypic spectrum associated with *EGFR* by demonstrating that a germline variant can underlie epilepsy and characteristic non-neoplastic MRI abnormalities. Our findings underscore the importance of multidisciplinary re-evaluation of variants of uncertain significance (VUS) and segregation analysis in large families.

## Introduction

Approximately 3% of the general population is affected by rare neurological disorders (1, 2).

Despite advances in exome and genome sequencing, a substantial proportion of cases remain unresolved, particularly when potentially pathogenic variants are classified as variants of unknown significance (VUS) (3). Interpretation of such variants often requires extensive phenotyping, segregation analysis, and multidisciplinary evaluation. However, as such approaches require substantial organizational and clinical effort, this time-consuming process is not routinely feasible, leaving many VUS unresolved.

The *EGFR* gene encodes a receptor tyrosine kinase that regulates growth, differentiation, maintenance, and repair of multiple tissues. Somatic pathogenic *EGFR* variants are well established in glioblastoma multiforme. Germline *EGFR* variants have mainly been associated with lung cancer, though only when combined with other (external) factors and/or additional somatic variants (4). Recently, an association between somatic *EGFR* variants and drug-resistant lesional focal epilepsy (LFE) caused by low-grade epilepsy-associated tumors (LEATs) was demonstrated (5).

Here, we describe a large family presenting with epilepsy, progressive cognitive decline, and highly characteristic cerebral MRI abnormalities, segregating with a rare germline *EGFR* variant initially classified as a VUS. Only after combined deep geno- and phenotyping in a multidisciplinary, multicenter setting, this variant was considered likely pathogenic. This case highlights the importance of systematic re-evaluation of genomic data in the context of detailed phenotyping and family-based analysis.

## Case description

### Summary of clinical and genetic findings

**Figure 1:**
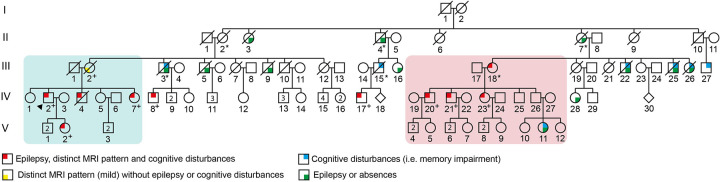
Pedigree of the family. ^+(plus)^ *EGFR* missense variant identified in ES genotyping data. ^*(asterisks)^ obligate carrier of the germline *EGFR* variant.

**Figure 2:**
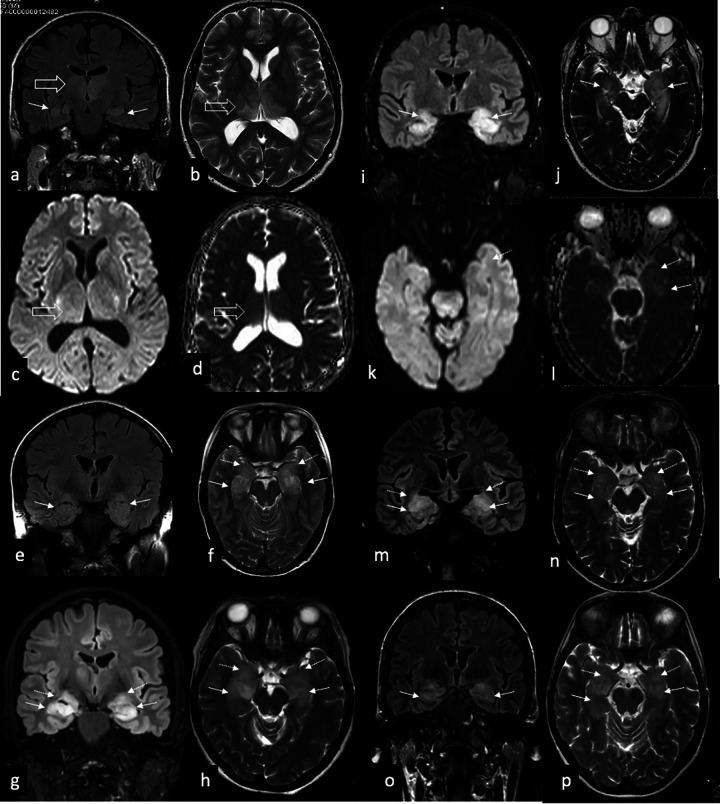
Similar MRI imaging findings in different patients:. a-d) index patient IV-1 with hyperintensity of the hippocampi on coronal FLAIR **(arrows, a)** and thalami on axial T2w [open arrow, **(b)** without restricted diffusion on DWI **(c)** and corresponding ADC **(d)** in the thalami e-f] coronal FLAIR **(e)** and axial T2w **(f)** of patient V-2 with bilateral hyperintensity and swelling of hippocampi (arrow) and amygdala (dotted arrow) g-h) coronal FLAIR **(g)** and axial T2w **(h)** of patient IV-7 with bilateral hyperintensity and swelling of hippocampi (arrow) and amygdala (dotted arrow) i-l) coronal FLAIR **(i)** and axial T2w **(j)** of patient IV-21 with hyperintensity and swelling of hippocampi (arrow) and amygdala (dotted arrow), left more than right without restricted diffusion in the mesial temporal lobe on DWI **(k)** and corresponding ADC **(l)** m-n) coronal FLAIR **(m)** and axial T2w **(n)** of patient IV-20 with bilateral hyperintensity and swelling of hippocampi (arrow) and amygdala (dotted arrow) o-p) coronal FLAIR **(o)** and axial T2w **(p)** of patient V-23 with bilateral hyperintensity and swelling of hippocampi (arrow) and amygdala (dotted arrow).

The index patient (IV-2, Figure 1; see also Table 1 for summary), a 60-year-old male, presented with unexplained syncope. Cardiological evaluation revealed a conduction disorder requiring pacemaker implantation. He was later admitted to the intensive care unit for hypercapnic coma, possibly related to obstructive sleep apnea, which was subsequently treated with continuous positive airway pressure (CPAP). From his admission onwards he developed temporal lobe epilepsy and progressive cognitive disturbances, predominantly memory impairment and reduced attention span. Neurological evaluation, including brain MRI, demonstrated rare, symmetrical bilateral T2-FLAIR hyperintensity and swelling of the mesiotemporal regions and thalami (Figure 2).

**Table 1:** Summary of clinical and genetic findings.

Individual	Age at symptom onset (years)	Key clinical features	Seizure classification	Brain MRI findings	Genetic testing and results	Follow-up/outcome
IV-2 (index patient)	60	Unexplained syncope; later temporal lobe epilepsy; progressive cognitive decline (memory and attention deficits); obstructive sleep apnea	Focal seizures with intact consciousness and sensory onset	Bilateral symmetrical mesiotemporal and thalamic T2-FLAIR hyperintensity with increased volume	Initial gene panels and exome sequencing negative; exome reanalysis identified heterozygous *EGFR* c.866C > A p.(Ala289Asp)	Persistent epilepsy and cognitive impairment; MRI abnormalities stable over time
V-2 (daughter of IV-2)	25	Temporal lobe epilepsy	Focal seizures with intact consciousness and sensory onset	Identical bilateral mesiotemporal and thalamic T2-FLAIR hyperintensity	Exome sequencing confirmed *EGFR* c.866C > A p.(Ala289Asp)	Ongoing epilepsy
IV-7	55	Drug-resistant temporal lobe epilepsy	Focal seizures with intact consciousness and sensory onset	Bilateral mesiotemporal abnormalities	Genetic reanalysis identified *EGFR* c.866C > A p.(Ala289Asp)	Seizure freedom after temporal lobe resection
IV-20	Adulthood	Temporal lobe epilepsy; cognitive impairment	Focal seizures with impaired consciousness and sensory onset	Bilateral mesiotemporal and thalamic T2-FLAIR hyperintensity	Trio-exome sequencing; *EGFR* c.866C > A p.(Ala289Asp) identified after reanalysis	Persistent epilepsy
IV-21	Adulthood	Temporal lobe epilepsy; verbal memory deficits	Focal seizures with intact and some with impaired consciousness and sensory onset	Bilateral hippocampal and thalamic abnormalities	Trio-exome sequencing; *EGFR* c.866C > A p.(Ala289Asp)	Persistent epilepsy and cognitive impairment
IV-23	Adulthood	Epilepsy	Focal seizures with impaired consciousness and sensory onset	Bilateral mesiotemporal and thalamic abnormalities	Trio-exome sequencing; *EGFR* c.866C > A p.(Ala289Asp)	Persistent epilepsy

Family history revealed that the index patient's brother (IV-4) died suddenly at the age of 34 years after a history of unexplained epileptic seizures since age 25. Autopsy revealed edema and gliosis/astrocytosis in the hippocampi and amygdalae. One of his sisters (IV-7) underwent right temporal lobe resection for drug-resistant epilepsy at the age of 55 years. Histopathological examination showed gliosis and atypical hippocampal sclerosis (ILAE type 3), without evidence of neoplasia.

Extensive diagnostic evaluation—including cerebrospinal fluid analysis, autoimmune antibody testing, muscle biopsy and DNA analysis using cardiomyopathy, arrhythmia, and mitochondrial gene panels as well as exome sequencing—did not identify a (likely) underlying cause.

During the diagnostic process, index patient's daughter (V-2) developed epilepsy at the age of 25 years. Brain MRI revealed identical bilateral mesiotemporal and thalamic abnormalities (Figure 2).

Upon suspicion of an autosomal dominant inherited disease, patients IV-2, V-2 and IV-7 were included in the ZOEMBA study.

In another hospital, three siblings (Family B: IV-20, IV-21, IV-23) presented with epilepsy and cognitive disturbances. Brain MR revealed increased bilateral mesiotemporal and thalamic T2 FLAIR signal intensity and volume (Figure 2). In all patients, MRI was performed using a 3 Tesla Philips Achieva or Ingenia Scanner, using an dedicated epilepsy, following the HARNESS guidelines (Table 2). Their verbal memory deficits could be directly connected to the hippocampal lesions in the dominant hemisphere. Upon suspicion of an inherited disease trio-exome sequencing was performed; however no (likely) pathogenic variants were identified. The *EGFR* variant detected by open exome analysis was at that time classified as VUS and not deemed causative.

**Table 2:** MRI scanning protocol for all patients.

MR sequence and plane	Voxel size (mm)	Repetition Time (ms)/Echo Time (ms)
T2 Axial	0.50 × 0.62 × 5.0	2,656/80
3D T1 Sagittal	1.0 × 1.0 × 1.0	811/3,708
IR Coronal	0.55 × 0.78 × 2.0	8,645/10
3D FLAIR Sagittal	1.10 × 1.11 × 1.20	8,000/337,94
DWI Axial	1.82 × 1.85 × 5.0	10,000/73,107
SWI Axial	0.6 × 0.6 × 2.0	

Additional family history taking in Family A subsequently revealed connection between Family A and Family B. Comparison of medical data demonstrated identical clinical phenotypes and strikingly identical and distinctive MRI abnormalities, supporting the clinical suspicion of a rare, novel autosomal dominant inherited disease.

### Diagnostic assessment and therapeutic intervention

Initial genetic testing did not identify pathogenic variants in any of the individual patients. Only after familial clustering of identical clinical symptoms and a characteristic MRI phenotype were recognized, suspicion of a rare, novel, likely autosomal dominant inherited disease arose. All affected individuals from Family A were therefore included in the international ZOEMBA study (http://www.clinicaltrials.gov; NCT06200142). This study focuses on deep phenotyping and reanalysis of exome sequencing data after standard diagnostic work-up has failed to yield a clinical diagnosis in a patient suspected of harboring an inherited disease.

In our patients, MRI showed symmetric T2- and FLAIR hyperintensities involving the mesiotemporal structures and thalami. The most common cause of symmetric involvement of the amygdala and hippocampus on MRI is limbic encephalitis. Limbic encephalitis, including both paraneoplastic and non-paraneoplastic forms such as those associated with GAD65 and GFAP antibodies, can also affect extra-limbic structures such as the striatum or thalami (6, 7). MRI in limbic encephalitis typically shows restricted diffusion due to cytotoxic edema (8), which was not observed in any of our patients. Moreover no detectable autoimmune antibodies were identified. In addition the abnormalities on T2-weighted and FLAIR images appeared solid rather than edematous. Limbic and thalamic involvement can also be observed in postictal changes, however these typically demonstrate restricted diffusion caused by excitotoxic edema and resolve on follow-up imaging (9). Bilateral thalamic involvement can also be seen in several toxic (exogenous) and metabolic (endogenous) pathologies, including Wernicke's encephalopathy, Leigh syndrome, flavivirus encephalitis and Creutzfeldt-Jakob disease; however, extensive clinical and genetic evaluation in our patients did not support any of these diagnoses. Moreover, these conditions are not consistent with the pattern of affected individuals observed in the pedigree, which suggests an autosomal dominant mode of inheritance.

As part of the ZOEMBA study, exome reanalysis was performed for affected individuals IV-2, V-2, and IV-7 using the Radboudumc custom annotation pipeline (10). Exome data (derived from patient's diagnostic BAM files) was filtered for rare variants (variant frequency <1% in dbSNP, GnomAD v2.1 and in-house database) that were shared by all three affected individuals. Short nucleotide variant calling was carried out using GATK v.3.4-46, copy number variants were called using Canvas Copy Number Variant Caller v.1.40.0 (Illumina), and variants were annotated using an in-house developed diagnostic pipeline. Short-tandem repeats (STRs) were analyzed using Expansion Hunter v3.1.2. with default settings. *De novo* mutations (DNMs) were called as previously described by Lelieveld et al. and by using DeNovoCNN v1.1.0 (10). Subsequently, missense variants with a CADD score >20 or SpliceAI score ≠ 0 and nonsense variants were prioritized resulting in a shortlist of 74 variants in 68 genes.

Because the anatomical distribution of the MRI abnormalities in our patients resembled the anatomical distribution seen in diffuse midline gliomas (11), genes associated with midline and temporal brain lesions were prioritized. This revealed a rare heterozygous *EGFR* [Chr7(GRCh38):g.55154129C > A/Chr7(GRCh37):g.55221822C] variant [NM_005228.5:c.866C > A; p.(Ala289Asp)]. The same variant was found in family B and reconsidered to underlie the phenotype in both families.

The *EGFR* variant is absent from GnomAD v4.1 which fulfills the ACMG PM2 criterion and provides supporting evidence for pathogenicity. The variant affects a highly conserved residue (phyloP score 7.882). Most *in silico* missense prediction tools suggested a deleterious effect [CADD score 29.7, REVEL score 0.590, AlphaMissense score 0.952, BayesDel (noAF) 0.26]. The Alanine residue at position 289 lies within the extracellular EGFR domain, a known hotspot for somatic gain-of-function variants in glioblastoma and other tumors (12, 13). SKMG03 is a human brain astrocytoma (glioblastoma) cell line that harbours the p.(Ala289Asp) variant in EGFR. Depletion of EGFR by shRNA induces cell death specifically in these cells, indicating that the p.(Ala289Asp) EGFR variant is the driver variant for SKMG03 cell proliferation and thus, a gain-offunction variant (14). This specific variant (‘A289D’) is listed as ‘Likely oncogenic’ and ‘Likely Gain of function variant’ in OncoKB database (15, 16) highlighting the functional relevance of this residue. We propose that this evidence supports application of ACMG criterion PM1 at supporting level as the variant is located in a mutational hotspot and/or critical, well-established functional domain, with six occurrences of A289D in Chang et al. (15).

Segregation analysis across both family branches demonstrated co-segregation of the *EGFR* variant with the neurological phenotype. One obligate carrier (III-2) remained largely asymptomatic at the age of 88 years, but showed milder MRI abnormalities. Of other obligate carriers, individual III-3 was reportedly clinically unaffected, while clinical details of II-2 were unavailable. However, subtle focal seizures with sensory onset (arising from the temporal lobe) may have gone unrecognized. Several additional family members (II-4, III-15, II-7, III-5, III-9, III-22, III-25, and III-26) were reported to have had comparable neurological symptoms, but detailed clinical data were no longer accessible. The co-segregation in this family, yielding a LOD score of 4.28, corresponding to a likelihood ratio of 19,03, provides evidence for pathogenicity under the ACMG PP1 criterion. According to the likelihood ratio thresholds proposed by Tavtigian et al. (17) this evidence would qualify as ‘Very Strong’. Since the co-segregation evidence is from a single family we formally cannot exclude the possibility of other, undetected, pathogenic variants in the same gene and therefore we propose to use PP1_strong.

Using the point based system (instead of combining criteria) a conservative classification (18) of *EGFR* c.866C > A p.(Ala289Asp) would be ‘Likely Pathogenic’ (PP1_strong, PM1_supporting, PM2_supporting). In clinical practice the classification of the variant would remain VUS (suspicious for pathogenicity) until the gene-disease association has been accepted (19).

### Follow-up and outcomes

Affected individuals showed persistent epilepsy and slowly progressive cognitive decline. MRI abnormalities remained stable over time, without radiological progression or development of neoplasia. Family history did not reveal an increased incidence of brain tumors or other malignancies.

Treatment consisted mainly of antiseizure medications. All affected family members are currently under shared care of a single neurologist to improve communication, continuity of care, and disease-specific expertise. Epilepsy was drug-resistant in several individuals.

## Discussion

This case report describes, for the first time, an association between a germline *EGFR* variant and epilepsy, progressive cognitive impairment and distinctive bilateral mesiotemporal and thalamic MRI abnormalities. Previously, *EGFR*-related epilepsy has only been reported in the context of somatic variants in lesional focal epilepsy (5).

In the adult brain, EGFR plays a critical role in regulating neurogenic niches in the hippocampus and thalamus (20). Increased EGFR expression has been observed in epileptogenic brain tissue, suggesting a role in epileptogenesis independent of neoplasia (21). Somatic EGFR mutations however promote tumorigenesis because they arise in a single proliferative cell and confer a clonal growth advantage under positive selection, often in cooperation with additional oncogenic alterations, leading to malignant expansion. For germline variants, being present in all cells from conception onwards, we hypothesize that these could potentially increase EGFR signaling promoting astrocytic activation and gliosis through upregulation of GFAP filaments, shifting astrocytes toward a reactive state (22, 23). This early imbalance in brain development between astrocytes and neurons may explain the glioma-like, yet non-neoplastic, MRI appearance and epilepsy observed in this family, particularly in regions rich in neural stem cells such as the hippocampus and thalamus (24).

Importantly, the genetic diagnosis only emerged after recognition that two independently investigated families with non-diagnostic exome sequencing and identical MRI abnormalities were in fact related, underscoring the critical role of deep phenotyping, extended family reconstruction, and periodic re-evaluation of VUS.

Strengths of this report include extensive phenotypic characterization and segregation analysis across a large multigenerational family, while limitations include the absence of functional validation studies.

### Patient perspective

Patients and family members described a prolonged and fragmented diagnostic trajectory with considerable uncertainty regarding prognosis and recurrence risk. Identification of a shared genetic cause provided clarity and facilitated genetic counseling, although targeted treatment options remain limited. Centralization of care under a single neurologist improved continuity of care and communication.

## Data Availability

The datasets presented in this article are not readily available due to privacy restrictions. Requests to access the datasets should be directed to the corresponding author.
